# Comparative Leaves Transcriptome Analysis Emphasizing on Accumulation of Anthocyanins in *Brassica*: Molecular Regulation and Potential Interaction with Photosynthesis

**DOI:** 10.3389/fpls.2016.00311

**Published:** 2016-03-18

**Authors:** Muhammad A. Mushtaq, Qi Pan, Daozong Chen, Qinghua Zhang, Xianhong Ge, Zaiyun Li

**Affiliations:** National Key Laboratory of Crop Genetic Improvement, National Center of Oil Crop Improvement (Wuhan), College of Plant Science and Technology, Huazhong Agricultural UniversityWuhan, China

**Keywords:** *Brassica*, pigmentation, transcriptome, anthocyanins, photosynthesis

## Abstract

The purple leaf pigmentation mainly associated with anthocyanins accumulation is common in *Brassica* but the mechanisms of its production and its potential physiological functions are poorly understood. Here, we performed the phenotypic, cytological, physiological, and comparative leaves transcriptome analyses of 11 different varieties belonging to five *Brassica* species with purple or green leaves. We observed that the anthocyanin was accumulated in most of vegetative tissues in all species and also in reproduction organs of *B. carinata*. Anthocyanin accumulated in different part of purple leaves including adaxial and abaxial epidermal cells as well as palisade and spongy mesophyll cells. Leave transcriptome analysis showed that almost all late biosynthetic genes (LBGs) of anthocyanin, especially *Dihydroflavonol 4-Reductase* (*DFR*), *Anthocyanidin Synthase* (*ANS*) and *Transparent Testa 19* (*TT19*), were highly up-regulated in all purple leaves. However, only one of transcript factors in anthocyanin biosynthesis pathway, *Transparent Testa* 8 (*TT8*), was up regulated along with those genes in all purple leaves, indicating its pivotal role for anthocyanin production in *Brassica*. Interestingly, with the up-regulation of genes for anthocyanin synthesis, Cytosolic 6-phosphogluconolactonase (*PLG5*) which involved in the oxidative pentose-phosphate pathway was up-regulated in all purple leaves and three genes *FTSH PROTEASE 8* (*FTS8*), *GLYCOLATE OXIDASE 1* (*GOX1*), and *GLUTAMINE SYNTHETASE 1;4* (*GLN1;4*) related to degradation of photo-damaged proteins in photosystem II and light respiration were down-regulated. These results highlighted the potential physiological functions of anthocyanin accumulation related to photosynthesis which might be of great worth in future.

## Introduction

Anthocyanins are water soluble pigment existing in many plants, algae, and bacteria. These are responsible for various color formation in leaves, flowers, stems, roots, and many other plant organs which usually attract pollinators and dispersers. Anthocyanins may also play roles in protecting chloroplast from the photo-oxidative and photo-inhibitory damage by scavenging free radicals and reactive oxygen species (ROS; Hughes et al., 2005; Hatier and Gould, 2008). Plants under stress conditions or infection by pathogens could also induce anthocyanins formation (Chalker-Scott, 1999; Lea et al., 2007; Kerio, 2011). Therefore, these pigments are highly essential for plant survival. Moreover, recent studies in tomato have indicated that accumulation of the anthocyanins on skin could double its shelf-life by delaying over-ripening and reducing the susceptibility to gray mold (Bassolino et al., 2013; Zhang et al., 2013, 2015b). A number of studies have suggested that the food with rich anthocyanins could benefit human health by its high antioxidant activity against cardiovascular disease, certain cancer, and some other chronic diseases (Hou, 2003; Butelli et al., 2008; Martin et al., 2011; Lila, 2004).

**Figure 1:**
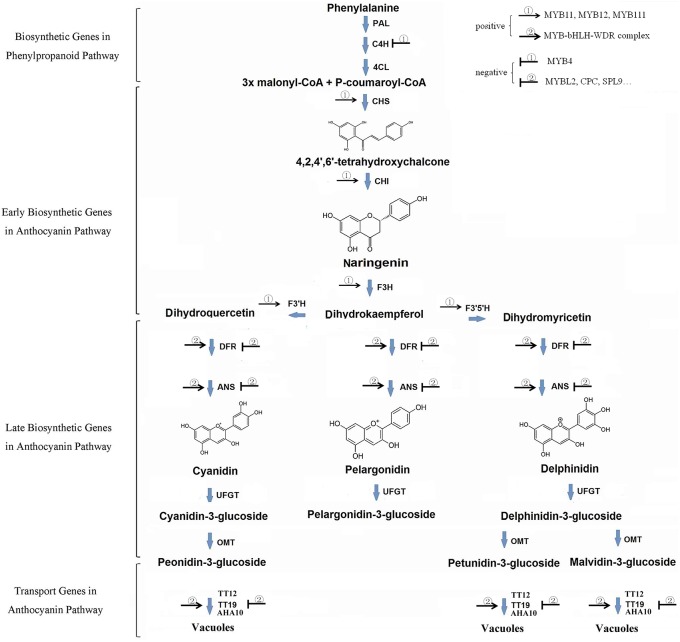
**Scheme of the biosynthetic pathway of anthocyanin**.

Anthocyanins are formed by phenylpropanoid metabolism from phenylalanine by series genes including early biosynthetic genes (EBGs) and late biosynthetic genes (LBGs) (Figure 1). Simply, three molecules Malonyl CoA and one of ρ-coumaroyl CoA are firstly condensed by chalcone synthase. The product 4, 2′4′6′-tetrahydrocychalcone are further catalyzed successively by four enzymes (Chalcone Isomerase [CHI], Flavanone 3-Hydroxylase [F3H], Dihydroflavonol 4-Reductase [DFR] and Anthocyanidin Synthase [ANS/LDOX]; Harborne and Grayer, 1994; Bohm, 1998; Dao et al., 2011). In addition to pelargonidin, two other anthocyanidin, cyanidin, and delphinidin, are formed in most plants by further hydroxylate the B-ring of dihydrokaempferol by Flavonoid 3′Hydroxylase [F3′H] and Flavonoid 3′5′ Hydroxylase [F3′5′H], respectively. Depending on variable cell environment, especially the vacuolar pH value, pelargonidin being orange to red, cyanidin being red to red-purple, and delphinidin being red-purple to blue. However, these pigments accumulate exclusively as glycosylated forms (anthocyanins) for the anthocyanidin structure are unstable. Till date, more than 600 natural anthocyanins have been identified which are derived from these core anthocyanins by methylated on its 3′ or 5′ or both hydroxyl group and side chain decorations, such as glycosylation and acylation (Glover and Martin, 2012).

The regulation of anthocyanin biosynthesis at transcription level is mainly operated by a series of transcript factors, especially for those R2R3-MYB genes. While early biosynthesis genes are active by co-activator independent and functionally redundant R2R3-MYB genes (e.g., MYB11, MYB12, and MYB111 in *Arabidopsis*), late biosynthesis genes are active by a highly conserved transcriptional activation complex MYB–bHLH–WDR (MBW) in angiosperms and likely also in gymnosperm (Figure 1; Hichri et al., 2011; Petroni and Tonelli, 2011; Xu et al., 2013, 2015). The complex consists of MYB proteins, basic Helix-Loop-Helix (bHLH) proteins, and a WD repeat protein. In *Arabidopsis*, R2R3-MYB genes *PAP1, PAP2, MYB113*, and *MYB114* (Borevitz et al., 2000; Gonzalez et al., 2008), *bHLH* gene family *TT8, GL3, EGL3* (Nesi et al., 2000; Payne et al., 2000; Zhang et al., 2003), and *WD40* family gene *TTG1* (Walker et al., 1999) are recognized as the key genes encoding respective components of MBW complex. In addition, various MYB TFs (i.e., MYBL2 and MYB4), SPL9 and LBD family genes are also reported as negative regulators of the anthocyanins accumulation (Jin et al., 2000; Dubos et al., 2008; Matsui et al., 2008; Rubin et al., 2009; Gou et al., 2011) (Figure 1).

Cultivated *Brassica* species belong to the monophyletic *Brassiceae* tribe within the dicotyledon family *Brassicaceae*, including three diploids *B. rapa* (AA), *B, oleracea* (CC), *B. nigra* (BB), and three allotetraploids *B. napus* (AACC), *B. juncea* (AABB), *B. carinara* (BBCC). Three allotetraploids are formed and evolved from the hybrids between any two of diploids (Nagaharu, 1935). Most of these species are important oilseed crops and vegetables worldwide. Although all *Brassica* species contain yellow flowers due to the accumulation of carotenoid (Zhang et al., 2015a), red and purple pigments are deposited at various part of *Brassica* plants especially at leaves, stems, and pods. Although a number of studies have been carried out in different *Brassica* species, the mechanisms behind such color formation are still poorly understood. In present study, we systemically investigated the pigments formation in five *Brassica* species and performed comparative transcriptome analysis between purple and green leaves. Our main objective was to examine potential key genes response for leaf pigmentation as well as the physiological roles of anthyocyanin accumulation in *Brassica* plants development.

## Materials and methods

### Plant materials, phenotypic characterization, and leaf anatomy observation

In this study, eleven varieties from five different *Brassica* species (*B. rapa, B. napus, B. juncea, B. oleracea*, and *B. carinata*) were used for phenotype observation, photosynthesis measurement, and RNA-seq analysis. For each species, except for *B. rapa*, one variety with purple and one with green leaves were investigated. For *B. rapa*, two type of varieties with purple and one with green leaves were used (Table S1). All varieties were planted in research field of Huazhong Agricultual University, Wuhan, China, during 2013–2014 cropping season. For each species, the purple and green varieties were cultivated side by side. Young leaves at seedling stage were collected at the same day for RNA extraction. Phenotypic comparison was carried out between purple and green for seed endosperms, young leaves, young seedlings of 6-days-old plants, and young plants of 6-weeks-old. To examine different types of cells showing purple pigmentation, Leaves were transversely sectioned by free hand and examined with a Zeiss Axioscope photomicroscope equipped with an MRC digital camera.

### Photosynthetic rate measurement

Photosynthesis can be measured by photosynthesis measurement systems. These systems measure the rate using an infrared gas analyzer to accesses the input of CO_2_ and output of H_2_O. In present study, portable photosynthesis machine LI-6400XT (LI-COR Inc., Lincoln, NE, USA) was used for recording photosynthetic rate in all five *Brassica* species. The gas exchange parameters were determined on sunny, windless days from 9:30 to 11:30 a.m. Leaf temperature was controlled at 12°C and photon flux density was maintained at 500 μmol m^−2^ s^−1^. Net photosynthetic rate (*A*), stomatal conductance (*g*s), and transpiration rate (*Tr*) were recorded on fully expanded leaves of second youngest nodes. The total three readings per treatment were taken from randomly selected plants.

### RNA extraction and mRNA-seq libraries preparation

Young leaves were collected with two biological replicates at seedling stage from the field and immediately frozen in liquid nitrogen and stored at −80°C until use. Total RNA was extracted using TRIzol (Invitrogen) according to the manufacturer's instructions. The quality of purified RNA was initially evaluated on agarose gel and then quantified using NanoDrop spectrophotometer (Thermo Fisher Scientific, Inc.). The integrity of RNA samples were further evaluated using an Agilent 2100 Bioanalyzer (Agilent Technologies, Inc.). The TruSeq TM RNA Sample Preparation Kit (Illumina, Inc.) was then used according to the manufacturer's instructions, to construct cDNA libraries. Concisely, poly-A mRNA was purified and fragmented into short fragments and used as templates for first strand cDNA synthesis. Then DNA polymerase I and RNase H were used to synthesize the second-strand cDNA. Purified short double strand cDNA fragments were connected with adapters (Illumina). Suitable ligated cDNA fragments were selected as templates for the PCR amplification for the finally library construction. Finally, the cDNA libraries were sequenced using Illumina HiSeq 2000 sequencing platform at National Key Laboratory for Crop Genetic Improvement in Huazhong Agricultural University, Wuhan, China.

### Processing of raw reads and mapping

Adaptors were removed from the reads firstly. The reads in which unknown bases comprised more than 5% of the total and low quality reads (the percentage of the low quality bases of quality value ≤ 5 is more than 50% in a read) were also removed. The clean reads were aligned to *B. napus* var. Darmor-*bzh* genome accessed from http://www.genoscope.cns.fr/brassicanapus/ allowing up to two mismatches in each segment alignment by Tophat (Trapnell et al., 2010) and Bowtier software. Only those unique mapped reads were used for further analysis.

### Assessment of differentially expressed genes (DEGs)

Cufflinks program was used to assemble aligned RNA-Seq reads into transcripts, to estimate their abundances, and to test for differential expression and regulation transcriptome-wide. The gene expression level and the transcripts abundances were calculated using FPKM method. If there were more than one transcript for a gene, the longest one was used to calculate its expression level and coverage. The significance of differential gene expression between the purple and green leaf *Brassica* species was determined using Cuffdiff (adjusted *p* ≤ 0.001 and Fold change ≥ 2 as criteria). Heat maps were prepared by HeMI software from the website: http://hemi.biocuckoo.org/faq.php.

### Gene expression analysis using semi qRT-PCR

To evaluate the validity of Illumina analysis and assess the expression profiles in terms of specific mRNA abundance, several genes were selected and detected by Semi qRT-PCR. Reverse transcription was performed by Super Script III Reverse Transcriptase (Invitrogen) and oligo (dT) according to the manufacturer's instructions. Forward and reverse primers were designed by using the Primer 3 software based on conserved sequence of the genes from different species or different copies within the same genome. Sequences of selected genes were obtained from the *B. oleracea* genome database: http://www.ocri-genomics.org/bolbase/index.html, *B. rapa* genome database: http://brassicadb.org/brad/ as well as *B. napus*: http://www.genoscope.cns.fr/brassicanapus/. Beta Actin, as the internal housekeeping gene control, was used to get the bands (25 cycle) using original cDNA. All the cDNA samples were diluted to a concentration which gives same bright bands using the actin primers. Then gene specific primers were used to get different bright bands from different materials (32 cycles). Amplification reactions were performed as the following: an initial denaturation step at 94°C for 5 min, 32 cycles at 94°C for 30 s, 55°C for 30 s, and 72°C for 30 s, a final extension at 72°C for 10 min and hold at 25°C. The electrophoresis gel run bands were analyzed to verify the specificity of Semi qRT-PCR. All primers used were list in Table S2.

## Results

### Accumulation of anthocyanins in different *Brasica* species

**Figure 2:**
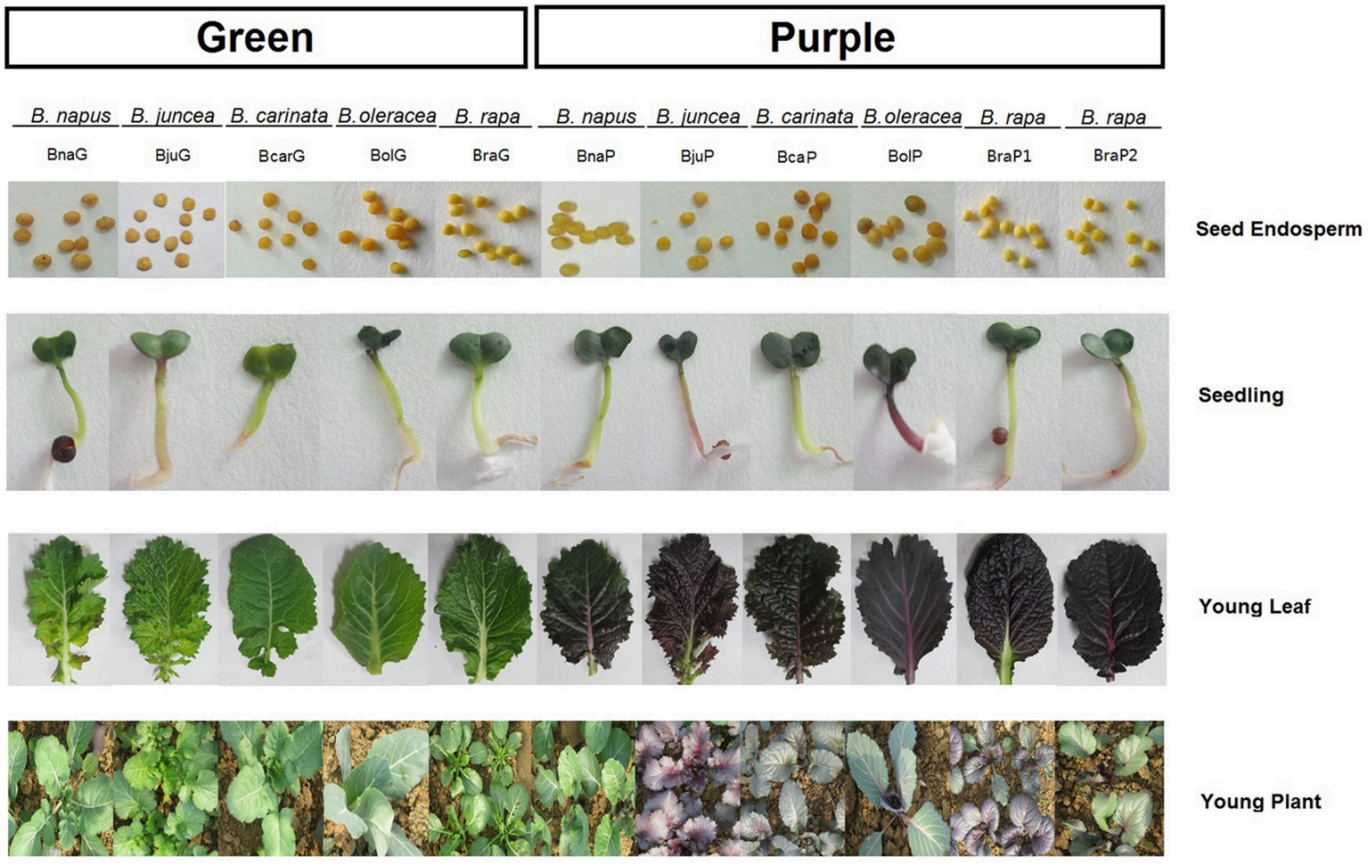
**Accumulation of anthocyanin at various tissues/organs of different *Brassica* species**.

All cultivars used in the study have yellow endosperms and dark seed coat except for BcaP which has a brown to yellow seed coat. Obviously, pigments do not accumulate in endosperms of these *Brassica* cultivars. The purple phenotypes seem to be related primarily with young seedlings, leaves, and young plants (Figure 2). The young seedlings of all green cultivars depicted green color except for BjuG with light red color but fade when true leaf was emerged. The young seedlings of BjuP and BolP showed dark red color, but other purple cultivars showed very light or no red color at this stage. During the first few weeks of growth, all the young leaves of green cultivars turned into solid green color, whereas those of purple cultivars displayed purple color in leaves (Figure 2). In later development stages, the anthocyanins were accumulated only on leaves in BjuP and BraP1, but in other species, the anthocyanins were found almost in all vegetative tissue of the plant, including petiole, stem, and flower stalk. However, only in BcaP, purple sepals and very little pigments on petals were apparent (data not shown). Under the identical growth conditions, the green plants showed no purple pigmentation in these tissues.

**Figure 3:**
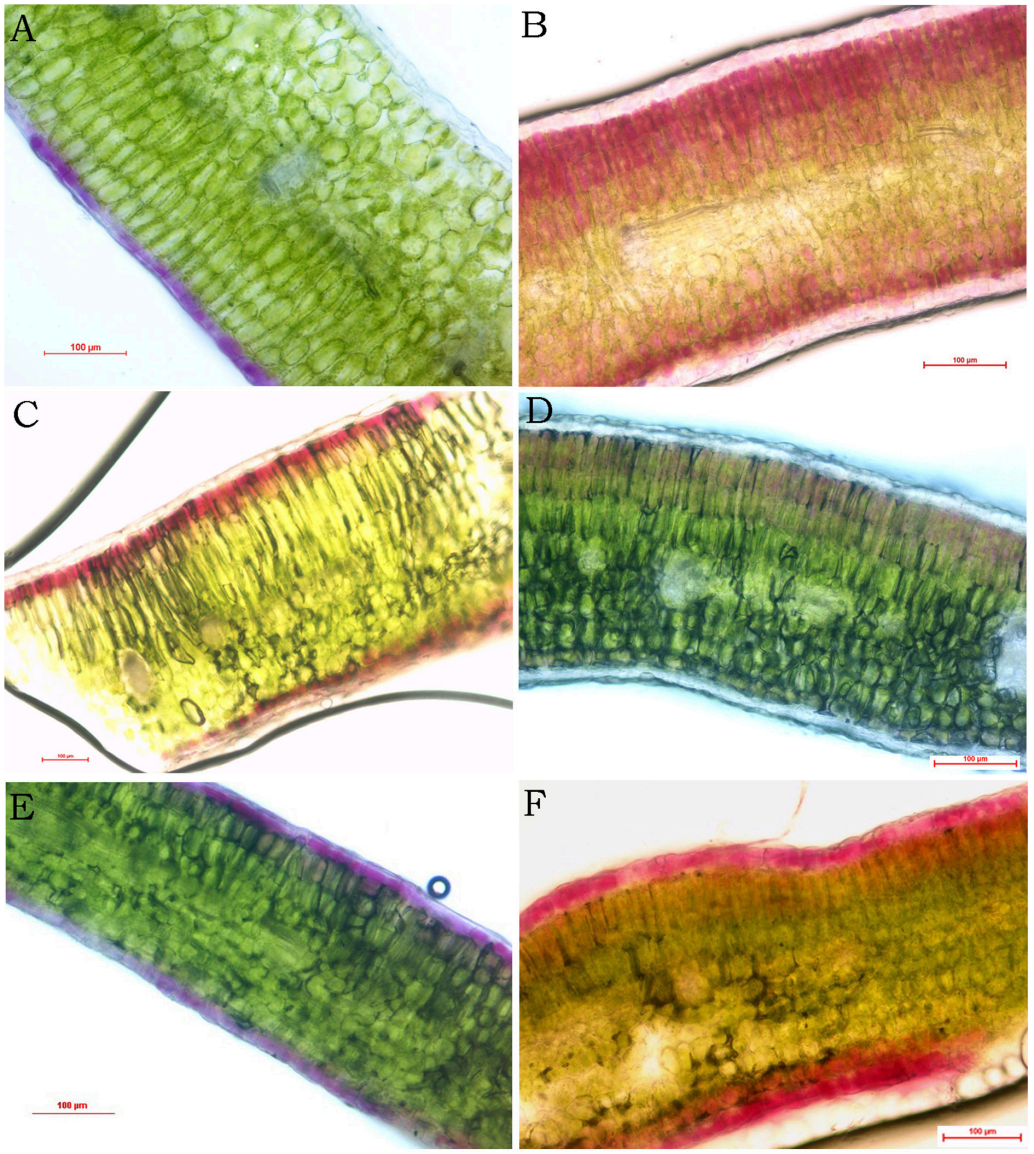
**Accumulation of anthocyanin in different part of purple leaves in *Brassica* revealed by hand section. (A–F)** purple leaves from *B. rapa* (BraP1), *B. rapa* (BraP2), *B. oleracea* (BolP), *B. napus* (BnaP), *B. juncea* (BjuP), and *B. carinata* (BcaP), respectively.

Utilizing free hand sectioning, the mounts of *Brassica* leaf transverse sections were observed. The results revealed that in BraP1, the distribution of purple pigmentation was solidly accumulated only in adaxial epidermal cells (Figure 3A). Contrary to BraP1, purple pigmentation was found only in mesophyll cells but not epidermal cells in leaves of BraP2 (Figure 3B). Interestingly, almost all palisade and spongy mesophyll cells have pigmentation with decreasing content from edge to center. In BolP, pigmentation was also accumulated in both palisade and spongy mesophyll cells but only in the first layer of the mesophyll cells (Figure 3C). In BnaP, purple pigmentation was not found in epidermal and spongy mesophyll cells but very light pigmentation was observed in palisade mesophyll cells (Figure 3D). In BjuP, pigmentation was accumulated in both adxial and abxial epidemal cells but almost noting in mesophyll cells (Figure 3E). In BcaP, dark purple pigmentation was observed in both adaxial and abaxial epidermal cells as well as in their adjacent one to several layers of palisade or spongy mesophyll cells (Figure 3F). In all five species, the leaves of all green cultivars did not show purple or red pigmentation in any cell type. In summary, the pattern of anthocyanin accumulation in *Brassica* leaves varies with species. BraP2 and BcaP showed more dark and wide distribution of pigments, while BnaP depicted the lightest purple pigmentation.

### Photosynthetic activity of leaves with different color of different species

**Figure 4:**
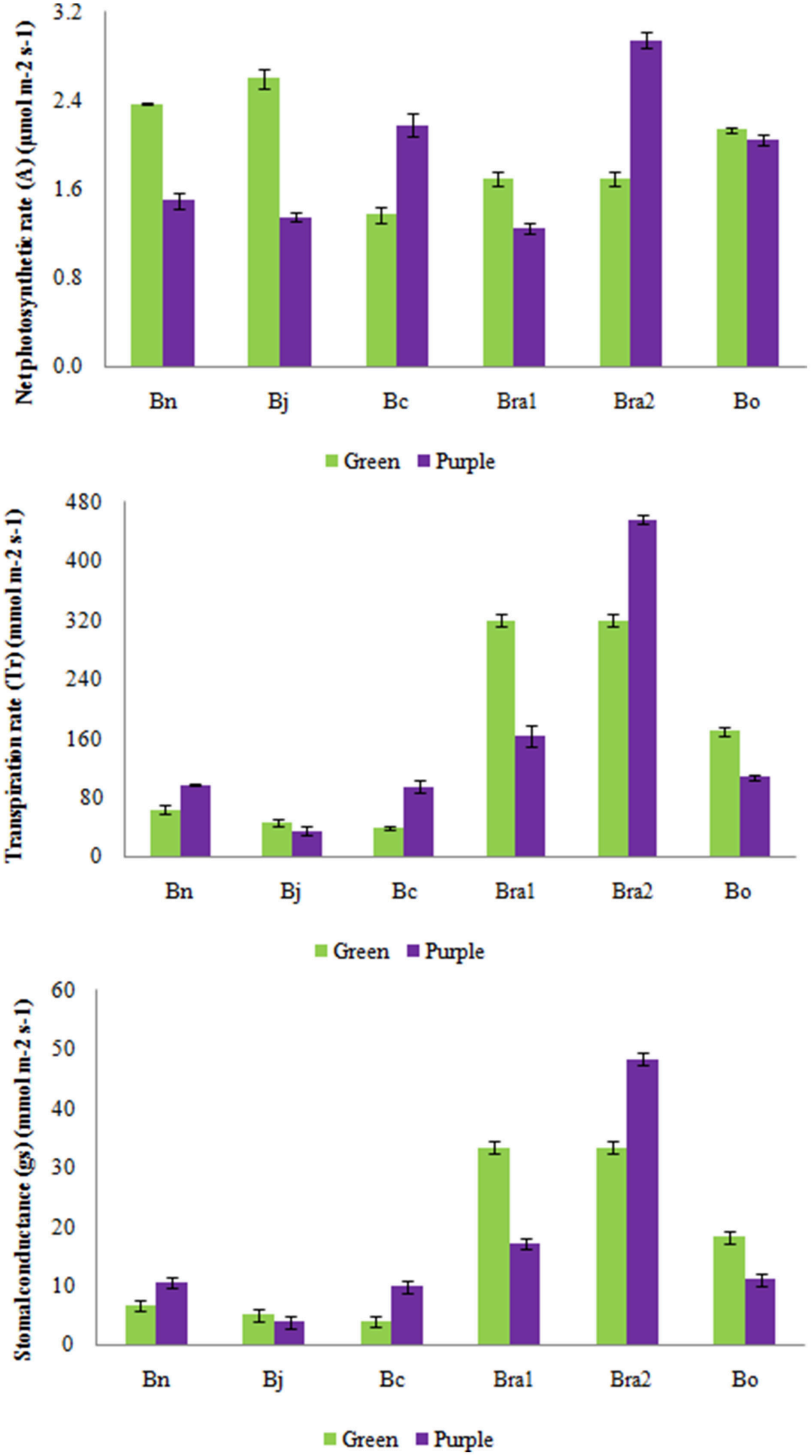
**Analysis of leaf stomatal conductance (gs), transpiration rates (Tr) as well as rates of photosynthesis (A) in green and purple leaves of different *Brassica* species**. Bn, *B. napus*; Bj, *B. juncea*; Bc, *B. carinata*; Bra1, BraP1 and BraG; Bra2, BraP2 and BraG; Bo, *B. oleracea*.

In order to investigate the potential effect of the anthocyanin accumulation on photosynthesis, we analyzed the photosynthesis-related attributes in all five *Brassica* species. Intraspecific studies revealed that stomatal conductance (*g*s), transpiration rates (*T*r) as well as rates of photosynthesis were higher in green leaves of *B. juncea, B. oleracea*, and *B. rapa* compared with their respective purple leaves (for *B. rapa* only BraP1; Figure 4; Table S3). Purple leaves of *B. carinara, B. rapa*P2 and *B. napus* recorded greater *g*s and *T*r than their green leaves, however, photosynthetic rate was higher only in BcaP and BraP2 (Figure 4; Table S3).

### Overall expression of genes in anthocyanin biosynthesis pathway of *Brassica*

From mRNA sequencing, raw data were obtained from two biological repeats of each purple and green leaves. In total, 498,148,526 raw reads were produced. After removing low quality reads, 455,173,884 clean reads were found (Table S4). More than 82.5% reads were mapped in *B. napus* genome from the *B. napus*, but only 37.9% in *B. juncea* and 40.6% in *B.carinata*. The reason might be that *B. carinata* and *B. juncea* have B genome, which is poorly associated with reference genome (A and C). On average, 85.6% mapped reads were unique mapped to the genome. According to each gene expression value (Supplementary Datasheet S1), the correlations of two duplicates were calculated and showed high repeatability in all species (Table S4). These results justified the high quality of sequencing data for further analyses.

**Figure 5:**
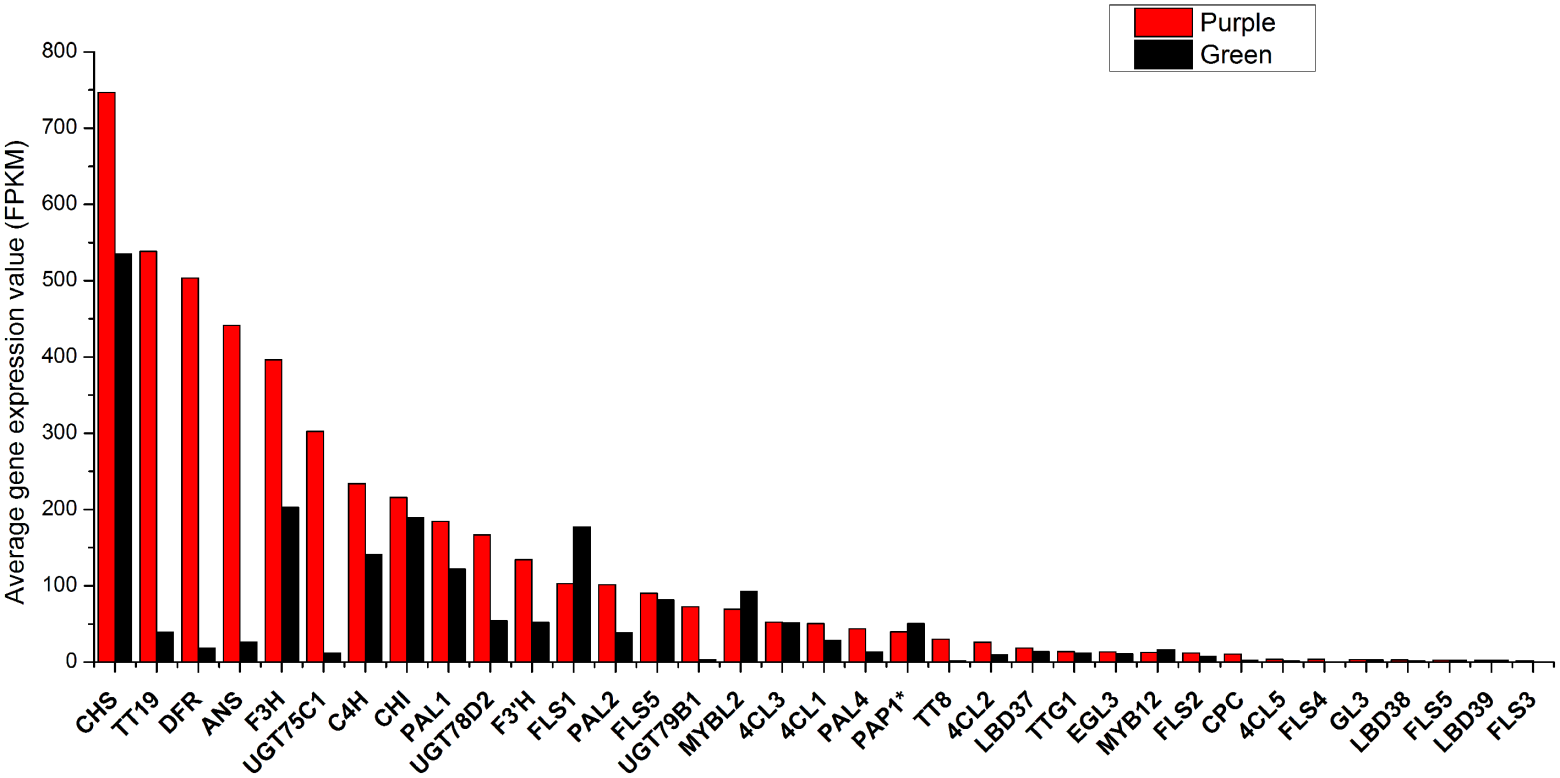
**Average total expression value (FPKM) of each gene in anthocyanin biosynthesis pathway in purple and green leaves of different *Brassica* species**.

Previously, 73 genes in *B. rapa* as orthologs of 41 anthocyanin biosynthetic genes in *A. thaliana* have been identified (Guo et al., 2014). Corresponding genes were identified in An and Cn sub-genome of *B. napus* according to http://brassicadb.org/brad/ respectively and their expression value were calculated (Supplementary Datasheet S2). It was found that *BrPAL3.1, BrPAL3.2, BrF3H2, BrTTG1.2, BrCHS5*, and *BrCHI3* as well as their syntenic genes in C genome are silenced in all materials (Supplementary Datasheet S2). To simplify analysis, for each gene in *Arabidopsis*, we added all its pralogous and orthologous genes in each *Brassica* species together for further analysis. Because it was difficult to discriminate *PAP1, PAP2, MYB113*, and *MYB114* at sequence level in *B. rapa* genome (Guo et al., 2014), PAP1^*^ was used to represent any of them in followed analysis. On average, 13 genes were expressed at high level (FPKM > 100) and most of which were LBGs, 14 genes expressed at middle level (FPKM = 10–100) and eight genes were expressed at low level (FPKM < 10) in purple leaves. In green leaves, only six genes were expressed at relative high level, including *CHS, CHI, F3H, FLS1, PAL1*, and *C4H*. Generally, most of the biosynthesis genes of anthocyanin in purple leaves were expressed at higher level than those in green leaves (Figure 5). Based on the expression of each gene, 11 varieties were classified into three main groups. All varieties with purple leaves except for *B. napus* were clustered into same group, while BnaP was grouped with BnaG, BjuG, and BraG (Figure S1). BolG and BcaG were divided into independent group. These results clearly showed that *B. carinata* and *B. oleracea* might have similar gene expression pattern while *B. napus* depicted a unique expression pattern of genes in anthocyanin synthesis.

### Differential expression of structural genes of anthocyanin biosynthesis pathway between purple and green leaves

**Figure 6:**
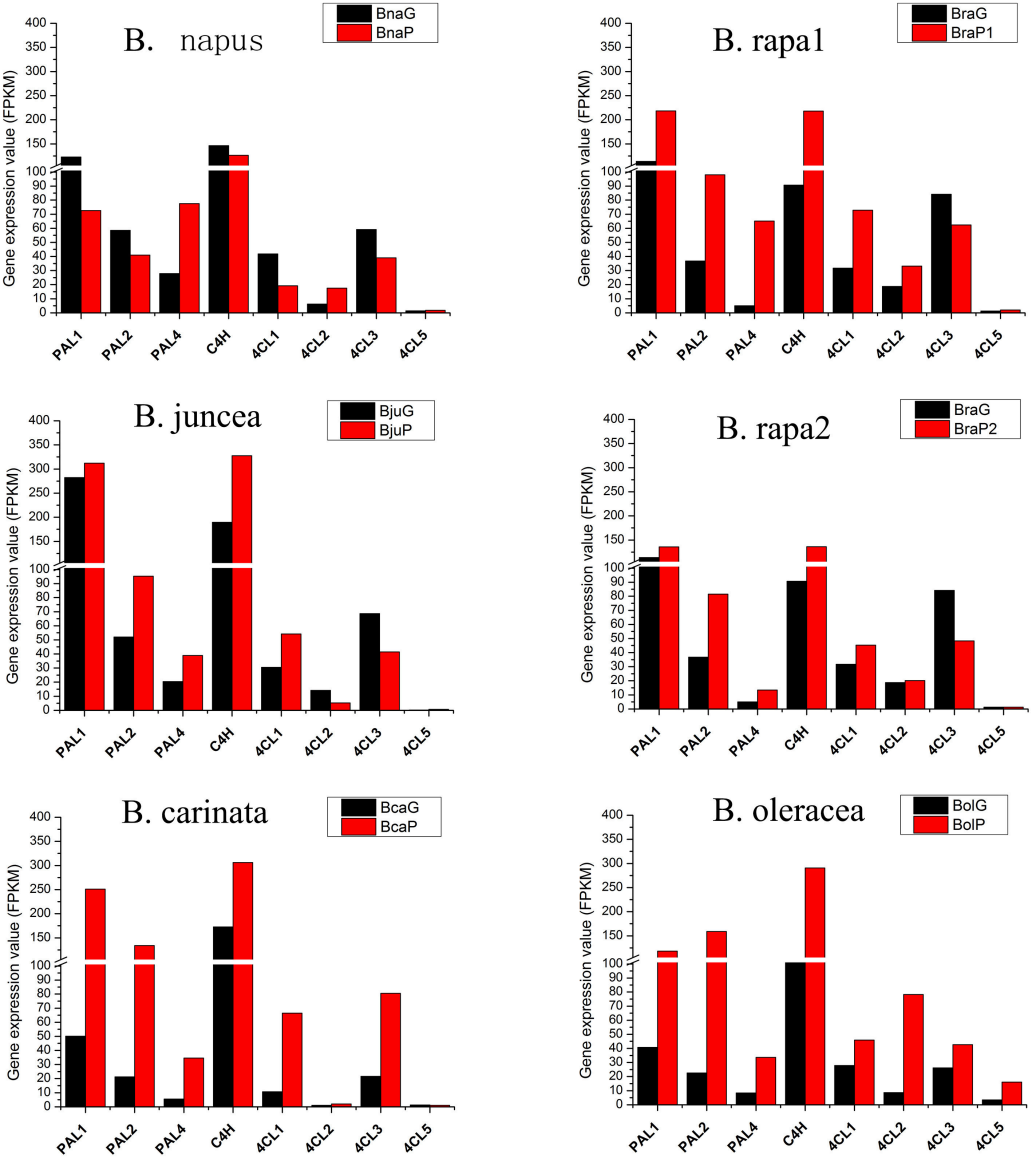
**Total expression value of structural genes in phenylpropanoid biosynthesis pathway in green and purple leaves of different *Brassica* species**.

**Figure 7:**
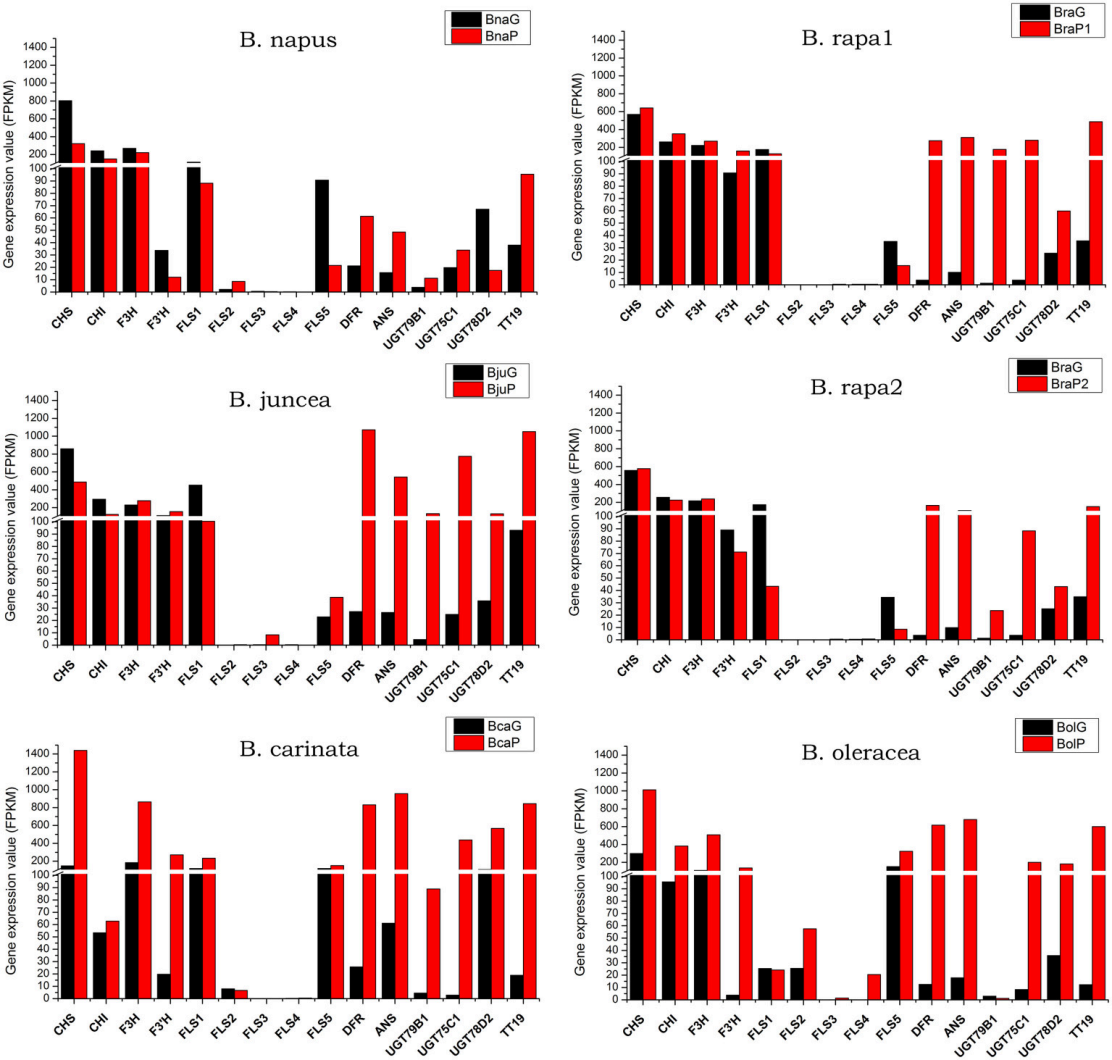
**Total expression value of structural genes in anthocyanin biosynthesis pathway in green and purple leaves of different *Brassica* species**.

Among eight genes in phenylpropopanoid metabolism, *4CL5* was expressed at very low level in all varieties and almost no difference was found between green and purple leaves, except for *B. oleracea*, where it was expressed at a little higher level in purple leaves (Figure 6). Other genes were highly expressed in purple leaves of *B. carinata* and *B. oleracea* and have higher expression level than those in respectively green leaves. In *B. rapa* and *B. juncea*, all other genes but *4CL3* were expressed at higher level in purple leaves than those in green leaves. However, only *PAL4* and *4CL2* were expressed at higher level in purple leaves than those in green leaves of *B. napus* (Figure 6). For EBGs, *CHS, CHI*, and *F3H* were expressed at much higher level in purple leaves of *B. carinata* and *B. oleracea* as well as a little higher level in BraP1, but showed similar level in green and purple leaves or lower level in purple leaves of other species (Figure 7). F3′H showed lowest expression in *B. napus* but much higher expression in *B. carinata* and *B. oleracea* in purple leaves. *FLS1* was expressed at lower level in purple leaves of all species except for *B. carinata*, where it showed a little higher expression. *FLS2, FL3*, and *FL4* were silenced or expressed at very low level (FPKM < 10) in all materials except for in *B. oleracea*. *FLS2* in *B. napus* and *B. oleracea, FL3* in *B. juncea*, and *FLS4* in *B. oleracea* showed higher expression in purple leaves. *FLS5* was down regulated in purples of *B. napus*, BraP1, and BraP2, but a little up-regulated in purple leaves of *B. oleracea, B. juncea*, and *B. oleracea*. *DFR, ANS* as well as *TT19* were greatly up-regulated in purple leaves of all varieties. Meanwhile, *UGT75C1, UGT79D2*, and *UGT78B1* depicted much higher expression in all purple leaves except for *UGT79D2* in *B. napus* and *UGT78B1* in *B. oleracea* where it was little down-regulated. In summary, EBGs of anthocyanin showed no prominent differences between green and purple leaves of *B. napus, B. juncea*, and *B. rapa*, but were highly up-regulated in purple leaves of *B. carinata* and *B. oleracea*. However, almost all LBGs, especially for *DFR, ANS*, and *TT19*, were highly up-regulated in all purple leaves.

### Differential expression of regulating genes of anthocyanin biosynthesis pathway between purple and green leaves

**Figure 8:**
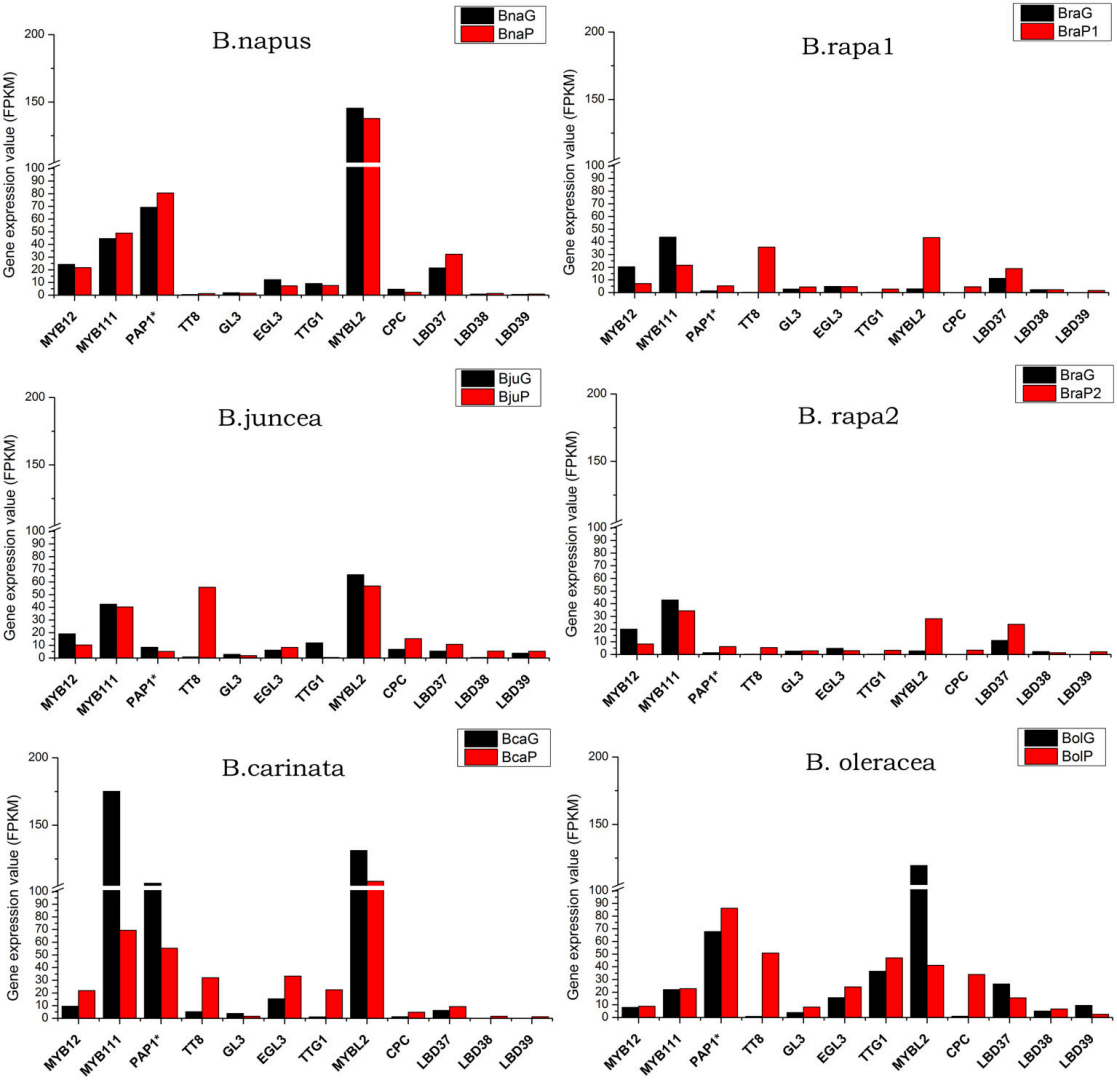
**Total expression value of regulator genes in anthocyanin biosynthesis pathway in green and purple leaves of different *Brassica* species**. PAP1^*^ represent any of PAP1, PAP2, MYB113, and MYB114.

It is well known that a series of transcript factor (TFs) play key roles in regulation of structural genes expression, and resulting in the accumulation of anthocyanin. In this study, all the TFs were expressed at relative low level in comparison with most of the structural genes. *MYB12* and MYB111 were only up-regulated in purple leaves of *B. carinata*, while down-regulated or showed no prominent differences between green and purple leaves of other varieties. PAP1^*^ (*PAP2, MYB113*, and *MYB114*), *TT8, TTG1*, and *EGL3* and *GL3* are the core proteins for MBW complex. It was found that *PAP1* was expressed at a little higher level in all purple leaves except for *B. juncea* and *B. carinata* (Figure 8). *GL3* and *EGL3* were expressed at very low level in all species except for *B. carinata* and *B. oleracea*, in which *EGL3* depicted relative higher expression in purple leaves. The *TT8* was the only positive TF which up-regulated in all purple leaves, especially in BraP2, *B. juncea, B. carinata*, and *B. oleracea* (Figure 8). For negative regulators, CPC was up-regulated at different degree in all purple leaves expect for *B. napus*, where it was down-regulated. MYBL2 was expressed at higher level in all leaves and was down regulated in purple leaves of *B. napus, B.carinata, B. oleracea*, and *B. juncea* but up-regulated in two *B. rapa*. *LBD 37, LBD38*, and *LBD39* were expressed at very low level. *LBD37* were little higher in purple leaves of all species except for *B. oleracea*. Although the expression pattern of *LBD* 38 and *LBD 39* was also similar in other purple and green leaves, *LBD38* in BcaG, *LBD39* in BcaG and BraG was silenced (Figure 8; Supplementary Datasheet S3).

**Figure 9:**
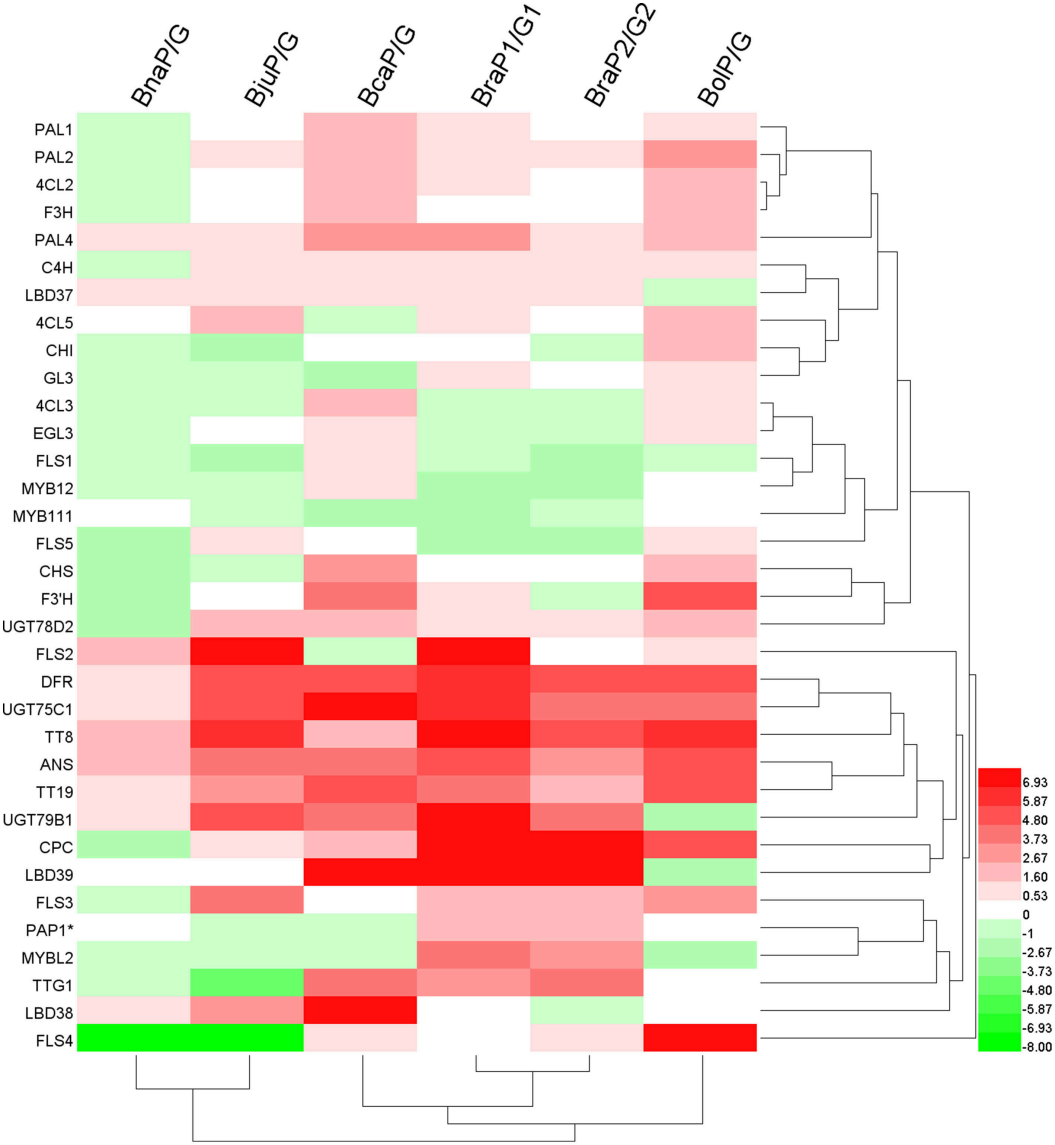
**Clustering analysis of all genes in anthocyanin biosynthesis pathway based on the fold changes between green and purple leaves**. Color bar: Log_2_ (fold changes). PAP1^*^ represent any of PAP1, PAP2, MYB113, and MYB114. Note: because some of genes were silenced (expression value = 0; Datasheet S3) in one of green or purple leaves, fold changes were set by hand according to the difference of each comparison.

In order to identify potential key regulators tightly related to anthocyanin accumulation in *Brassica*, we performed clustering analysis of all genes and varieties based on gene expression changes (fold change) between purple and green leaves (Figure 9). While two *B. rapa, B. napus* and *B. juncea* were clustered separately into two groups, *B. oleracea* and *B. carinata* occupied relative independent branches. This result indicated that two types of *B. rapa, B. juncea* and *B.napus* might have similar mechanism respectively for anthocyanin accumulation in leaves. Most of structural genes were clearly clusters into two major subgroups, one included genes involved in phenylpropopanoid pathway and EBGs and the other included all of LBGs (Figures 1,9). Meanwhile, transcription factors involved in MBW complex were grouped with those LBGs except for GL3 and EGL3. Interestingly, some of the transcripts were grouped into independent subgroups, for example, EGL3, MYB11 and MYB111, CPC and LBD38, *PAP1, LBD38, TTG1*, and *MYBL2*. These results are well consist with that there are complex mutual regulating relationships between different transcript factors in anthocyanin synthesis (Petroni and Tonelli, 2011). Prominently, *DFR, ANS, TT19, UGT75C1, UGT79B1*, and only transcript factor, *TT8*, are cluster together prominently, indicating that expression changes of *TT8* has major effect on the expression of LBGs for anthocyanin accumulation in *Brassica* leaves.

### RT-PCR validation of gene expression in anthocyanin biosynthesis pathway

To verify the relationships of gene expression revealed by RNA-seq analysis between green and purple leaves, a total of nine genes from anthocyanin biosynthetic pathway were chosen to perform semi-quantitative RT-PCR. Because these primers were designed based on the conserved sequence of each gene, the results will reflect the total expression values other than from specific copies. The relative transcript levels in all purple and green *Brassica* species were compared with those in RNA-seq data. In *B. oleracea*, qRT-PCR analysis showed that all genes but *PAL1* has the same expression trends as those in RNA-seq data (Figure S2A; Figures 6–8). In other four species, seven genes were performed RT-PCR analysis while *PAP1* and *CHS* cannot work well. Most of these genes also have the same expression trends as those in RNA-seq data, especially for *TT8* and *MYBL2* (Figure S2B; Figures 6–8). These results further confirmed the reliability of RNA-seq data in present study.

### CO-DEGs analysis between purple and green leaves of different *Brassica* species

In order to analyze the potential effect of anthocyanin accumulation on other gene expression, we firstly analyzed the differentially expressed genes between purple and green varieties and identified same DEGs by comparing the varieties with purple and green leaves. It was found that *B. oleracea* had the largest number of DEGs (6582) while *B. rapa 2* has the smallest number of DEGs (1057) (Supplementary Datasheet S4; Table S5). In order to analysis common DEGs in all species, we converted gene ID of *B. napus* into gene ID of *Arabidopsis*, because 11 varieties are belonging to five species with different genome. Finally, 21 common DEGs were found in all comparisons. Most of these genes have roles in photosynthesis, anthocyanin synthesis, and ribosome components (Table S6). We then calculated total expression value of these genes in each variety (Supplementary Datasheet S5). It was noteworthy, that *PLG5* which encodes a cytosolic 6-phosphogluconolactonase and thought to be involved in the oxidative pentose-phosphate pathway (OPPP), was highly expressed in all purple leaves along with *TT19, ANS*, and *DFR*. Meanwhile, four genes are co-down regulated in varieties with purple leaves, including *FTS8, GOX1, GLN1*, and *COR27* (Table S6). *FTS8* encode FtsH protease that is localized to the chloroplast. GOX1 encodes a glycolate oxidase that is the key genes in light respiration and modulates reactive oxygen species-mediated signal transduction during non-host resistance. *GLN;1.4* encodes a cytosolic glutamine synthetase, and takes part in assimilation process of the ammonia produced by the light respiration and the reduction of nitric acid. However, there is no function information of *COR27* till date.

## Discussion

With the rapid decrease in cost, trascriptome analysis using RNA-seq technology have become one of the most frequent and reliable methods for gene identification, genome evolution, developmental regulation, and genetic mapping studies. The available genome for *B. rapa, B. oleracea* and *B. napus* greatly facilitated the transcriptome analysis in *Brassica* species, especially for homeologous genes identification (Wang et al., 2011; Chalhoub et al., 2014; Liu et al., 2014; Parkin et al., 2014). Here, we performed comparative transcriptome analysis between different varieties belonging to five species with purple and green leaves. Using the *B. napus* as reference genome, we emphasized on the expression of the genes related with anthocyanin synthesis in *B. rapa* (Guo et al., 2014). Although each variety with purple and green leaves has different genetic background, we are focusing only on those similar results in different comparisons which will provide us credible conclusion.

### Leaf pigmentation and genome relationships in *Brassica*

Variations in leaf pigmentation are common in *Brassica* especially those used for vegetables, for example, red cabbage and purple cauliflower in *B. oleracea* and “Hongshancaitai” in *B. rapa* in China, but limited information is available in *B. nigra*. Because *Brassica* tetraploids are evolved from the hybrids between pair of diploids, it is reasonable to suspect that the variation of leaf pigmentation in tetraploids might be due to variations in corresponding parental diploids. Clustering analysis based on the expression of genes for anthocyanin synthesis in different species also reflected their genome relationships. The varieties with purple leaves were in same group except for *B. napus*. Meanwhile, BraP1, BraP2, and BjuP were occurred in the same group and BolP and BcaP were classified into other group (Figure S2). However, free hand section analysis showed that the distribution of pigmentation in leaves varies with species (Figure 3). The pattern of pigmentation in leaves of tetraploids was different from those in diploids. It looks that the variation in tetradploids was occurred independently from those diploids. An excellent example is that the *B. napus* with purple leaves used here is one of the progenies from wide hybridization between *B. napus* and *O. violaceus* (Ge et al., unpublished data). This *B. napus* have a different distribution of pigmentation in leaves and a very special expression pattern of those genes response for anthocyanin biosynthesis (Figures 2,3,9; Figure S1).

### TT8 plays key roles in regulating leaf pigmentation in *Brassica*

Many studies had been done to find key genes response for pigmentation in *Brassica* species at gene expression level. *BoMYB2* and *BrTT8* were two regulators identified by mapping based cloning for pigmentation in curds of *cauliflower* (*Brassica oleracea* var *botrytis*) and seeds of *Brassica rapa*, respectively till now (Chiu et al., 2010; Li et al., 2012). In this study, comparative transcriptome analysis between paired green and purple leaves of 11 varieties belonging to five different species clearly showed that the most of anthocyanin biosynthesis genes were greatly up-regulated, especially for those LBGs (Figure 7). These results indicated that anthocyanin accumulation in different part of leaves of different species might results from the variation of regulators involved in MBW complex which regulating LBGs. While other components of MBW are up or down regulated in purple leaves, *TT8* was up-regulated along with LBGs in all purple leaves. Particularly, the expression values of *TT8* generally appear positive relationships with the anthyocyanin content (Figures 2,3, 8). Our results were in accordance with previous studies investigated the gene expression by RT-PCR and suggest that TT8 was highly up-regulated in all *Brassica* with purple tissues and appeared to be a key candidate gene (Yuan et al., 2009; Xie et al., 2014; Zhang et al., 2014, 2015c; Ahmed et al., 2015).

In *Arabidopsis*, the main components of the MBW complexes are WD40, bHLH and MYB transcription factors. WD40 transcription factor is encoded by *TTG1*, bHLH are encoded by *TT8, GL3* and *EGL3* and MYB are encoded by *PAP1, PAP2, MYB113*, and *MYB114* (Borevitz et al., 2000; Ramsay and Glover, 2005; Gonzalez et al., 2008). It was found that in *Arabidopsi*s TT8 was required for the full transcriptional activation of late biosynthesis genes (Nesi et al., 2000) although it exhibits partially functional redundancy with *GL3* and *EGL3* (Zhang et al., 2003). *GL3* and *EGL3* contribute equally to the activation of *F3*′*H*, but *EGL3* appears more predominant in activation of *DFR* and *ANS* in Arabidopsis seedling pigmentation (Gonzalez et al., 2008). Here, *GL3* and *EGL3* expressed at very low level and have far clustering relationships with late biosynthesis genes (Figure 9). *TT8* thus might the key bHLH factor in MBW complex for leaves pigmentation in *Brassica* species. A complex regulation network among these regulators had been described (Zhang et al., 2003). *TT8* appears to be positively regulated by an MBW complex including the WD40 (TTG1), the MYB (TT2, PAP1/PAP2/MYB113/MYB114), and the bHLH itself or GL3/EGL3 and negatively regulated by MYBL2. Meanwhile, it also positively regulated TT2, PAP1/PAP2/MYB113/MYB114, GL3/EGL3, and MYBL2 (Baudry et al., 2004; Gonzalez et al., 2008; Petroni and Tonelli, 2011). Two modules were found to specifically drive *TT8* promoter activity by differentially integrating the signals issued from different regulators in a spatiotemporal manner which involves at least six different MBW complexes (Xu et al., 2013). These complex regulation relationships might make it difficult to indentify linear dependence between any two regulators as found here. For example, while *TT8* was upregulated in all purple leaves, *MYBL2* is upregulated in two *B. rapa* but down regulated in other purple leaves (Figure 8).

### Potential function of anthocyanin accumulation in leaves in *Brassica*

Although anthocyanins exist commonly in different species, their function in plant environment interactions remain highly contested. Till date, four putative functions of anthocyanins were proposed in plant development including (1) sunscreens and antioxidants, (2) mediators of reactive oxygen species (ROS)-induced signaling cascades; (3) anthocyanins may serve as metal-chelating agents under conditions of excess edaphic metal ions; (4) delayers of leaf senescence, especially in plants growing under nutrient deficiency (Landi et al., 2015). The first two types of functions were thought to be tightly related to photosynthesis. Because anthocyanin absorb strongly in the blue-green waveband, thus effectively reduce the wavelengths and intensity of light available to be used for photosynthesis (Nicole, 2009). Meanwhile, under high-light condition the down-regulation of internal light by anthocyanin plays key roles in photoprotection (Nicole, 2009). Additionally, as strong antioxidant, anthocyanin could protect tissues against radical oxygen species (ROS) generated in the chloroplast (Manetas, 2006).

The accumulation of anthocyanins within leaf tissues varied significantly among species or among different varieties of same species. Usually, anthocyanins are accumulated and stored in cell vacuoles, in or just below the adaxial epidermis, but sometimes these pigments entangled in photo-protection, and also accumulate in cell vacuoles of the abaxial epidermis, palisade and spongy mesophyll cells of leaves (Hatier and Gould, 2009). Our results showed that photosynthetic rate was higher in purple leaves of *B. carinata* and B. raP2 but lower in purple leaves of other species (Figure 4). Meanwhile, hand section analysis showed that high content of anthocyanin was absolutely in mesophyll of purple leaves in *B. rapa* P2 and both in epidemical and mesophyll cells in *B. carianta* (Figures 2, 3). Although anthocyanin was also distributed in mesophyll cells in purple leaves of *B. napus* and *B. oleracea*, but either the content is very lower in *B. napus* or only in limited layer of mesophyll cells in *B. oleracea*. It looks that high content of anthocyanin located in mesophyll might promote photosynthesis. However, this needs more evidence to make confirmation because the materials used here have very different genetic background. And many studies in other species had been shown that leaves with accumulation of anthocyanin have less photosynthesise than green leaves (Gould et al., 2002). So, comparison analysis between purple and green leaves with same genetic background would help to explore this question in *Brassica* in future (Tohge et al., 2005).

Interestingly, we found that 21 same DEGs between green and purple leave of all species, which potentially results from the accumulation of anthocyanin in purple leaves (Figure 9). These included *PLG5, FTS8, GOX1, GLN1;4*, and *COR27* which is co-upregulated or co-downregulated in all purple leaves. PLG5 is a key enzyme in oxidative pentose-phosphate pathway (OPPP) which produces many intermediate products including phenylalanine, the precursor for anthocyanin synthesis. The upregulation of *PLG5* in purple leaves are well with that anthocyanin accumulation there. FtsH belongs to subfamily ATPases Associated with diverse cellular Activities (AAA+). In plants, FtsH exists as a heterocomplex comprising isomers of two types: FtsH5/FtsH1 (Type A) and FtsH2/FtsH8 (Type B) (Yu et al., 2004, 2005; Zaltsman et al., 2005a). A series of studies indicated that thylakoid-embedded FtsH degrades photo-damaged D1 reaction center proteins in the photosystem II (PSII) repair cycle (Kato and Sakamoto, 2010; Nixon et al., 2010) and to control chloroplast development (Lindahl et al., 2000; Bailey et al., 2002; Sakamoto, 2003; Zaltsman et al., 2005a,b). Because studies had showed that foliar anthocyanins can protect chloroplasts from the adverse effects of excess light (Pietrini et al., 2002; Steyn et al., 2002; Close and Beadle, 2003; Hughes and Smith, 2007; Gould et al., 2010), anthocyanins accumulated in *Brassica* purple leaves might absorb a fraction of the yellow/green and ultraviolet wavelengths, and thus reduce the damage to PSII, and in particular that related to D1 repair and the oxygen evolving complex (OEC; Landi et al., 2015). These might lead to the down-regulation of *FstH8* in purple leaves. GOX1 and GLN1;4 are two important enzyme in plant photorespiration. *GOX1* encodes a glycolate oxidase catalyzes the conversion of glycolate into glyoxylate during photorespiration with concomitant production of H_2_O_2_ in peroxisomes (Foyer and Noctor, 2009). *GLN1;4* encodes a cytosolic glutamine synthetase, take part in assimilation process of the ammonia produced by the light respiration in chloroplast and the reduction of nitric acid. Simultaneously down-regulated of *GOX1* and *GLN1;4* in all purple leaves indicated that the photorespiration might be repressed in purple leaves in *Brassica*. Similarity, in the *Arabidopsis chi/f3*′*h* mutant that does not accumulate anthocyanins, the expression levels of *GOX1* were lower than in the wild type (Zhou et al., 2013). In summary, although we did not find direct relationships between the photosynthesis rate and the accumulation of anthocyanin in leaves of *Brassica* species, it looks that purple pigments play roles in reducing the damage to PSII and repressing photorespiration which were reflected at gene expression level.

## Conclusion

We performed comprehensive gene expression analysis related to anthocyanin biosynthesis along with phenotype and physiological observations in different *Brassica* species with purple and green leaves. Our results depicted that anthocyanin was accumulated in different parts of the leaves in different *Brassica* species, which might be due to the variations in core components of MBW complex. Particularly, TT8 might play pivotal role for the regulation of leaf pigmentation because it was the only up-regulated transcript factor in all purple leaves. Meanwhile, genes involved in OPPP pathway and light respiration were also found co-up or co-down regulated accompanied with the accumulation of anthocyanin in purple leaves which indicated the possible interaction between anthocyanin and photosynthesis.

## Author contributions

The study was conceived by XG. MM prepared the plant materials and performed the experiments. QZ performed sequencing. MM, QP, and XG performed the bioinformatics analysis. MM, XG, and ZL prepared the manuscript. All authors contributed to revising the manuscript. All authors had read and approved the final manuscript.

## Availability of supporting data

All the sequencing data used in this research has been submitted to public database NCBI under PRJNA298501 (accession number: SRP064721).

### Conflict of interest statement

The authors declare that the research was conducted in the absence of any commercial or financial relationships that could be construed as a potential conflict of interest.

## Supplementary material

Figure S1 **Clustering analysis of all genes in anthocyanin biosynthetic pathway based on total expression value between green and purple leaves**. PAP1^*^ represent any of PAP1, PAP2, MYB113, and MYB114.

Figure S2 **RT-PCR analysis of some genes in anthocyanin biosynthesis pathway**.

Table S1 **All materials used in this study**.

Table S2 **Primers used for RT-PCR analysis in this study**.

Table S3 **Analysis of leaf stomatal conductance (gs), transpiration rates (Tr) as well as rates of photosynthesis (A) in green and purple leaves of different *Brassica* species**.

Table S4 **Number of raw and clean reads, their mapping and repeatability analysis**.

Table S5 **The numbers of DEGs between green and purple leaves of different species**.

Table S6 **Functional annotation of co-DEGs between purple and green leaves of different species**.

Supplementary Data Sheet S1 **Expression value of each gene identified in different samples in different *Brassica* species**.

Supplementary Data Sheet S2 **Expression value of each gene in anthocyanin biosynthesis pathway**.

Supplementary Data Sheet S3 **Total expression value and fold changes between green and purple leaves of genes in biosynthesis pathway of anthocyanin**.

Supplementary Data Sheet S4 **Analysis of differentially expressed genes between green and purple leaves**.

Supplementary Data Sheet S5 **Total expression value of 21 co-DEGs between purple and green leaves in different *Brassica* species**.

## Abbreviations

PAL: Phenylalanine ammonia-lyase
C4H: Cinnamate-4-hydroxylase
4CL: 4-coumarate:CoA ligase
CHS: Chalcone synthase
CHI: Chalcone Isomerase
F3H: Flavanone 3-Hydroxylase
F3′H: Flavonoid 3′Hydroxylase
F3′5′H: Flavonoid 3′5′Hydroxylase
FLS: Flavonol synthase
DFR: Dihydroflavonol 4-Reductase
ANS: Anthocyanidin Synthase
TT19: Transparent Testa 19/Glutathione Transferase
TT8: Transparent Testa 8
TT2: Transparent Testa 2
UFGT: UDP glucose-flavonoid 3-O-glucosyltransferase
TFs: Transcription Factors
MBW: MYB–bHLH–WDR
bHLH: basic Helix-Loop-Helix
WDR: WD repeat
PAP1: Production of Anthocyanin Pigment 1
PAP2: Production of Anthocyanin Pigment 2
EGL3: Enhancer of Glabrous 3
TTG1: Transparent Testa Glabrous 1
GL3: Glabrous 3
CPC: CAPRICE
MYBL2: MYB-Like 2
LBD: LOB Domain-containing Protein
SPL9: SQUAMOSA PROMOTER BINDING PROTEIN-LIKE 9
PGL5: Cytosolic 6-Phosphogluconolactonase
OPPP: Oxidative Pentose-Phosphate Pathway
PSII: Photosystem II
GOX1: Glycolate Oxidase
GLN1: Glutamine Synthetase
COR27: Cold Regulated gene 27
ROS: Reactive Oxygen Species
LSD: Fisher's Least significant difference
A: Net photosynthetic rate
gs: Stomatal conductance
Tr: Transpiration rate
DEGs: Differentially Expressed Genes
FPKM: Fragments per Kilobase of exon per Million fragments mappedapped
EBG: Early biosynthesis genes
LBG: Later biosynthesis genes
OMT: *O*-methyltransferase family protein
GST: Glutathione S-Transferase
PCR: Polymerase Chain Reaction.
