# Supplementary material for: Comparative Leaves Transcriptome Analysis Emphasizing on Accumulation of Anthocyanins in Brassica: Molecular Regulation and Potential Interaction with Photosynthesis
Source: Front Plant Sci. 2016 Mar 18;7:311. doi: 10.3389/fpls.2016.00311 (PMC4796009; doi:10.3389/fpls.2016.00311)
Supplement: Figure S1 — Clustering analysis of all genes in anthocyanin biosynthetic pathway based on total expression value between green and purple leaves. PAP1* represent any of PAP1, PAP2, MYB113, and MYB114. [file Image1.PDF]

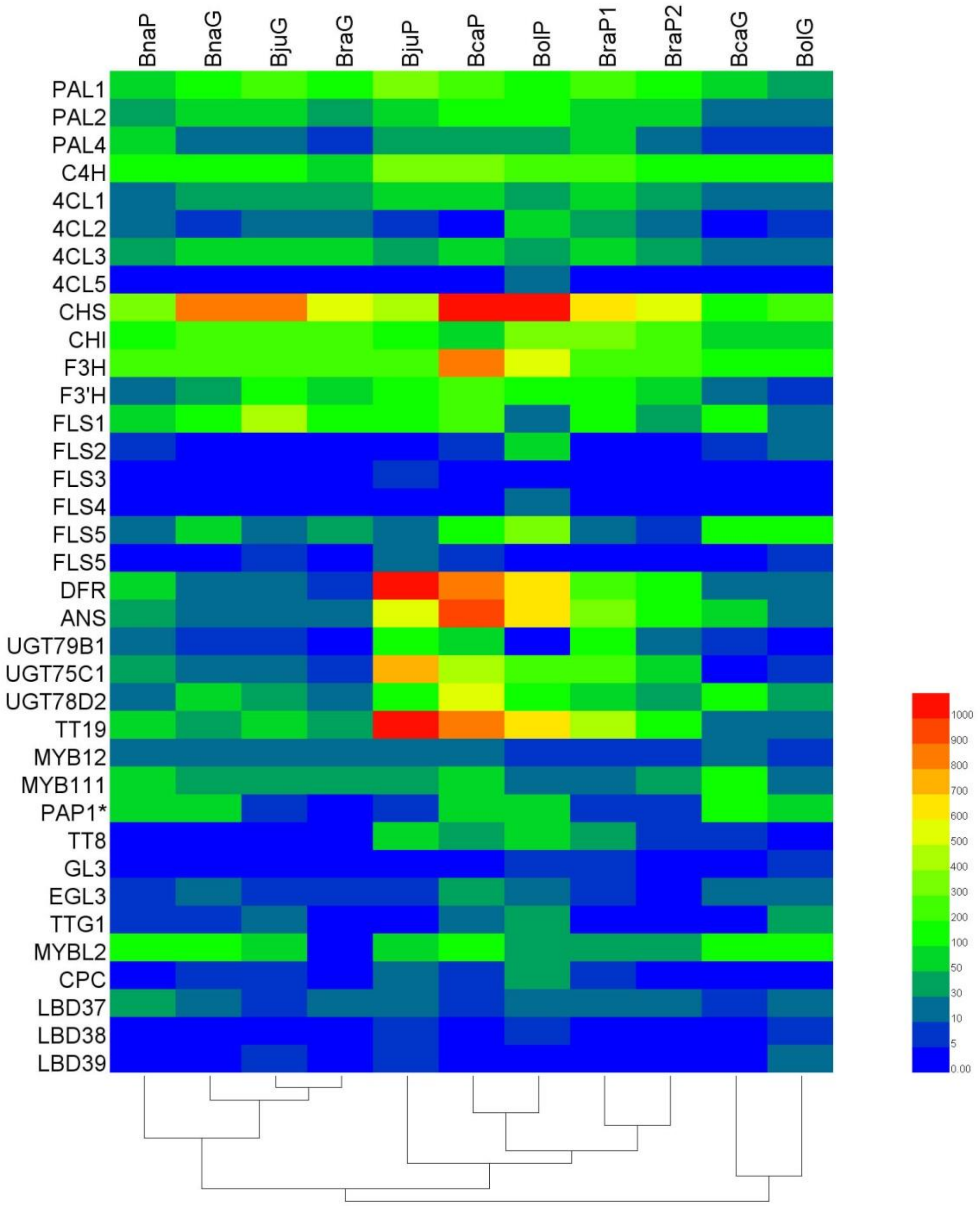

Figure S1. Clustering analysis of all genes in anthocyanin biosynthetic pathway based on total expression value between green and purple leaves. PAP1* represent any of PAP1, PAP2, MYB113 and MYB114.
