# Supplementary material for: Comparative Leaves Transcriptome Analysis Emphasizing on Accumulation of Anthocyanins in Brassica: Molecular Regulation and Potential Interaction with Photosynthesis
Source: Front Plant Sci. 2016 Mar 18;7:311. doi: 10.3389/fpls.2016.00311 (PMC4796009; doi:10.3389/fpls.2016.00311)
Supplement: Figure S2 — RT-PCR analysis of some genes in anthocyanin biosynthesis pathway. [file Image2.PDF]

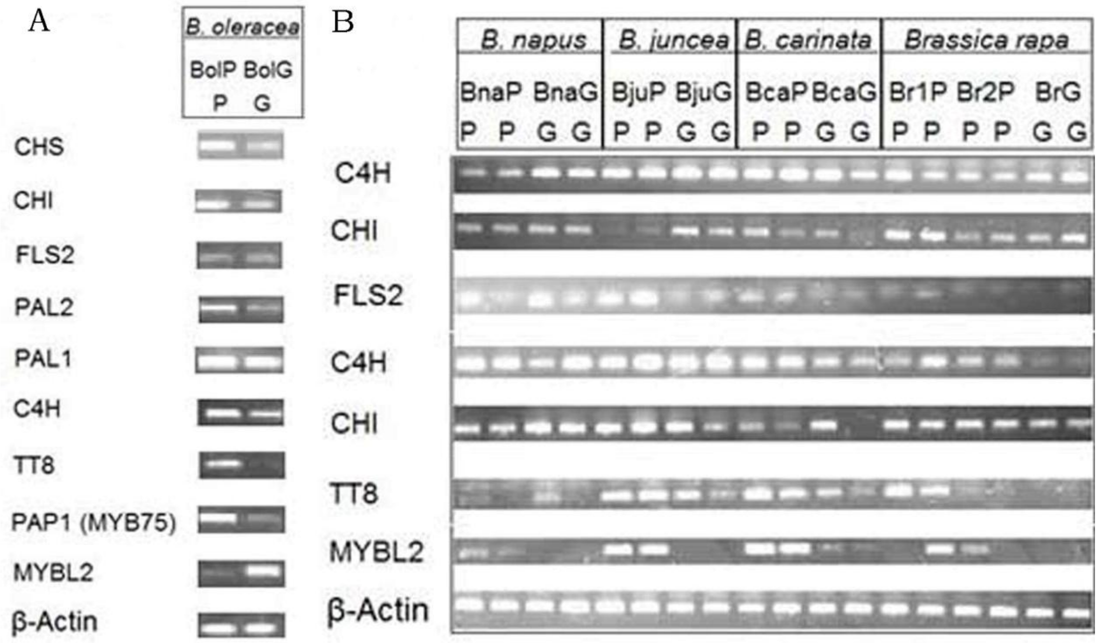

Figure S2. RT-PCR analysis of some of genes in biosynthetic pathway of anthocyanin. A: RT-PCR analysis in *B. oleraeca*; B: RT-PCR analysis in *B. napus*, *B. juncea*, *B. rapa* and *B. carinata*. P: purple leaves; G: green leaves.
