# Supplementary material for: Comparative Leaves Transcriptome Analysis Emphasizing on Accumulation of Anthocyanins in Brassica: Molecular Regulation and Potential Interaction with Photosynthesis
Source: Front Plant Sci. 2016 Mar 18;7:311. doi: 10.3389/fpls.2016.00311 (PMC4796009; doi:10.3389/fpls.2016.00311)
Supplement: Table S1 — All materials used in this study. [file Table1.pdf]

Table S1. All materials used in this study

Species	Leaf color	Genotype/accession number	Subspecies/Crop Type	Symbol
1. *Brassica rapa*	Purple	Zibaicai	*Brassica campestris*	BraP1
		Hongshancaitai	*Brassica campestris var*. purpuraria	BraP2
	Green	Kenshi	*Brassica chinensis*	BraG
2. *Brassica oleracea*	Purple	Xinhongmu	*Brassica oleracea var. capitata*	BolP
	Green	02-12	*Brassica oleracea var. capitata*	BolG
3. *Brassica napus*	Purple	GZ-1	Semi-winter type oilseed	BnaP
	Green	Zhongshuang11	Semi-winter type oilseed	BnaG
4. *Brassica juncea*	Purple	Ziyejiecai	*Brassica juncea var. gracilis*	BjuP
	Green	G19		BjuG
5. *Brassica carinata*	Purple	*CGN1159*		BcaP
	Green	*CGN1148*		BcaG

Table S2. Primers used for RT-PCR analysis in this study

Gene	5' to 3' Forward Primer	5' to 3' Reverse Primer
C4H	GTCGCTGCGATTGAGACAAC	TCAGGCTCTGTGACTTGCAC
CHS	GCGATCCTTGACGAGGTTGA	CGTCGTAGCCACACCATCTT
CHI	TGACGATGAAGCTGCCGTTA	AGCGAAGAGGATGGATGCAC
FLS2	AGGCGATCCGATCTGGAGAA	CCATCTCCTGTGTGCTTCGT
PAL2	CGACGGAAGCTCGTACATGA	CCACTGAGGAGAGGTACGGA
PAL1	CAACGTTCCTCGTCGCAATC	AAGCTCACCGTTGACTCCAG
TT8	AATCATCGATGGAAGCGAGG	TCATTTGCACCACTTAGCCA
PAP1(MYB75)	CAAACCTCGACCTCGATCCT	CACCACCTATTCTGACCATCC
MYBL2	TCGATCCAACCAATCATCGT	CCGGCACTAGCATTATCCTC

Table S3. Analysis of stomatal conductance (gs), transpiration rates (Tr) as well as rates of photosynthesis (A) in green and purple leaves of different *Brassica* species.

**Parameters**	**Net photosynthetic rate (A)**		**Transpiration rate (Tr)**		**Stomal conductance (gs)**	
**Units**	(µmol m-2 s-1)		(mmol m-2 s-1)		(mmol m-2 s-1)	
**Species**	Green	Purple	Green	Purple	Green	Purple
Brassica napus	2.37 ±0.01	1.50 ±0.06	63.57 ±5.77	97.34 ±0.72	6.67 ±0.01	10.59 ±0.00
Brassica juncea	2.60 ±0.08	1.35 ±0.04	46.91 ±5.01	35.67 ±5.64	4.96 ±0.01	3.76 ±0.01
Brassica carinata	1.37 ±0.07	2.18 ±0.10	39.26 ±2.26	95.04 ±7.62	3.97 ±0.00	9.91 ±0.01
***Brassica rapa* P1**	1.70 ±0.06	1.26 ±0.05	319.96 ±8.42	163.42 ±14.27	33.42 ±0.01	17.14 ±0.01
***Brassica rapa* P2**	1.70 ±0.06	2.95 ±0.07	319.96 ±8.42	456.28 ±5.98	33.42 ±0.01	48.36 ±0.01
Brassica oleracea	2.14 ±0.03	2.05 ±0.05	169.93 ±5.22	108.31 ±3.61	18.20 ±0.01	11.10 ±0.00

Each value represents mean of three replicates ± SE.

Table S4. Number of raw and clean reads, their mapping and repeatability analysis

Species	Duplicates	Raw data (Reads)	Clean data (Reads)	Total mapping (%)	Unique mapping (%)	Correlation
Brassica rapa	BraP1	22,171,200	20,541,872	13,970,509 (68.0%)	1,743,791 (87.5%)	0.953
		18,931,588	17,257,064	11,731,300 (68.0%)	1,518,199 (87.1%)	
	BraP2	20,820,232	18,418,448	12,380,693 (67.2%)	1,617,280 (86.9%)	0.968
		20,370,406	18,869,378	14,270,175 (75.6%)	6,524,279 (54.3%)	
	BraG	20,289,124	18,677,082	12,784,027 (68.4%)	1,865,614 (85.4%)	0.904
		23,051,574	21,252,826	14,584,961 (68.6%)	2,001,536 (86.3%)	
Brassica oleracea	BolP	26,056,356	23,143,932	17,975,319 (77.7%)	1,721,556 (90.4%)	0.948
		22,334,628	19,813,850	15,383,179 (77.6%)	1,438,140 (90.7%)	
	BolG	21,463,016	19,128,136	15,135,419 (79.1%)	1,385,988 (90.8%)	0.982
		22,538,906	20,236,346	15,980,813 (79.0%)	1,422,355 (91.1%)	
Brassica napus	BnaP	21,962,496	20,219,780	15,987,677 (79.1%)	1,963,988 (87.7%)	0.949
		21,371,738	19,528,750	16,097,511 (82.4%)	2,345,557 (85.4%)	
	BnaG	20,990,744	19,231,610	16,080,161 (83.6%)	1,429,387 (91.1%)	0.968
		18,996,786	16,946,838	14,378,037 (84.8%)	3,410,995 (76.3%)	
Brassica juncea	BjuP	19,870,912	17,946,950	6,753,251 (37.6%)	945,825 (86.0%)	0.971
		19,870,954	18,000,086	6,752,109 (37.5%)	921,925 (86.3%)	
	BjuG	24,859,152	22,910,184	8,591,360 (37.5%)	1,261,017 (85.3%)	0.978
		23,296,300	21,409,578	8,349,106 (39.0%)	1,208,934 (85.5%)	
Brassica carinata	BcaP	22,087,954	20,445,210	8,725,425 (42.7%)	1,164,107 (86.7%)	0.977
		23,494,160	21,688,268	9,085,315 (41.9%)	1,221,769 (86.6%)	
	BcaG	31,100,796	29,155,214	11,281,457 (38.7%)	1,307,780 (88.4%)	0.887
		32,219,504	30,352,482	11,971,610 (39.4%)	1,332,432 (88.9%)	

Table S5. The numbers of DEGs between green and purple leaves of different species

Species	Gene numbers		
	Up	Down	Total
B. napus(P/G)	3126	2859	5985
B. juncea(P/G)	2314	4401	6715
B. carinata(P/G)	589	937	1526
B. rapa R1(P/G)	487	570	1057
B. rapa R2(P/G)	575	1243	1818
B. oleracea(P/G)	3614	2968	6582

Table S6. Functional annotation of co-DEGs between purple and green leaves of all species

	Arabidopsis Gene ID	Functional Annotation
Co-down regulation in all varieties with purple leaves	AT1G06430*	FTSH8, encodes a FtsH protease that is localized to the chloroplast
	AT3G14420*	GOX1, Encodes a glycolate oxidase that modulates reactive oxygen species-mediated signal transduction during nonhost resistance.
	AT5G16570	GLN1;4, Encodes a cytosolic glutamine synthetase, the enzyme has high affinity with substrate ammonium
	AT5G42900	COR27, cold regulated gene 27 (COR27); BEST Arabidopsis thaliana protein match is: unknown protein (TAIR:AT4G33980.1);
Co-UP regulation in all varieties with purple leaves	AT5G17220	TT19, Encodes glutathione transferase belonging to the phi class of GSTs.
	AT5G42800	DFR, dihydroflavonol reductase. Catalyzes the conversion of dihydroquercetin to leucocyanidin in the biosynthesis of anthocyanins
	AT4G22880	ANS, encodes leucoanthocyanidin dioxygenase, which is involved in proanthocyanin biosynthesis.
	AT5G24420	PGL5, Encodes a cytosolic 6-phosphogluconolactonase (PGL) thought to be involved in the oxidative pentose-phosphate pathway (OPPP).
Others	AT1G09690	Translation protein SH3-like family protein; FUNCTIONS IN: structural constituent of ribosome;
	AT1G29930	LHCB1.3 Subunit of light-harvesting complex II (LHCII),which absorbs light and transfers energy to the photosynthetic reaction center
	AT2G28000	CPN60A, Encodes chaperonin-60 alpha, a molecular chaperone involved in Rubisco folding.
	AT2G33380	PXG3, Encodes a calcium binding protein whose mRNA is induced upon treatment with NaCl, ABA and in response to dessication.
	AT2G34430	LHCB1.4, Photosystem II type I chlorophyll a/b-binding protein
	AT3G08610	Unknown protein, mitochondrial respiratory chain complex I
	AT3G27690	LHCB2.3, Encodes Lhcb2.4. Belongs to the Lhc super-gene family encodes the light-harvesting chlorophyll a/b-binding (LHC) proteins that constitute the antenna system of the photosynthetic apparatus.
	AT4G16980	Arabinogalactan-protein family; INVOLVED IN: biological_process unknown; LOCATED IN: endomembrane system;
	AT4G19840	ATPP2-A1, encodes a phloem lectin, similar to phloem lectin in

	cucumber and celery.
AT4G39260	GRP8, Encodes a glycine-rich protein with RNA binding domain at the N-terminus.
AT5G02380	MT2B, cysteine-rich protein with copper-binding activity
AT5G16250	Unknown protein, Located in chloroplast
AT5G59910	HTB4, HTB4; FUNCTIONS IN: DNA binding; INVOLVED IN: nucleosome assembly; LOCATED IN: nucleolus
